# Thematic Analysis and Mapping of Reproductive Empowerment Scales: A Tool for Family Planning Self-Care Programming and Research

**DOI:** 10.9745/GHSP-D-21-00794

**Published:** 2022-06-29

**Authors:** Holly M. Burke, Reana Thomas

**Affiliations:** a FHI 360, Durham, NC, USA.

## Abstract

Understanding the relationship between self-care and reproductive empowerment is necessary to improve family planning self-care interventions. We offer a thematic analysis of 5 validated scales measuring reproductive empowerment-related concepts that could be used by self-care family planning programmers and researchers.

## Key Messages

- There is an evidence gap in the relationship between self-care and reproductive empowerment; understanding this relationship using validated scales is necessary to improve the design, implementation, and scale-up of family planning self-care interventions.
- We summarize 5 validated scales that measure reproductive empowerment-related concepts and offer a thematic analysis to help family planning programmers and researchers assess which scales best fit their needs for family planning self-care programming.
- Communication, decision making, and partner influence are the most measured domains among the scales we examined; however, measures of other reproductive empowerment domains are available and may be of interest to programs depending on their context and focus.

## INTRODUCTION

In the past decade, the field of family planning has seen an acceleration in conversations about self-care interventions and, within that, a resurgence of discussions on reproductive empowerment. The World Health Organization (WHO) defines self-care as^1^:

*the ability of individuals, families, and communities to promote health, prevent disease, maintain health, and cope with illness and disability with or without the support of the health provider.*

Self-care spans a range of practices including self-awareness, self-testing, and self-management, and self-care interventions are tools that support self-care.^1^ Reproductive empowerment is defined as the capacity of individuals to achieve their reproductive goals,^2^ and it is recognized as a fundamental principle for self-care.

Understanding the relationship between self-care and empowerment is necessary to improve the design, implementation, and scale-up of family planning self-care interventions. Yet, evidence on the relationship between reproductive empowerment and self-care, including the strength of the association, the direction of the association, or the hypothetical causality, is inadequate. A recent systematic review we conducted to understand the relationship between contraceptive self-care interventions and reproductive empowerment found clear gaps in the gray and published literature.^3^ Following the WHO guideline on self-care interventions,^1^ certain user-dependent methods were always included (e.g., oral contraceptive pills, condoms, rhythm method), whereas other interventions were included in certain circumstances (e.g., contraceptive injectables when self-injected, fertility awareness tools including digital apps, urine pregnancy tests when used for initiating a family planning method). Client-facing digital technologies were included if they (1) were accessible by clients with or without a health care provider; and (2) were created to provide individualized information, guidance, or self-management of contraception to enhance access, acceptability, use of and/or intention to use contraception. The vast majority of the existing evidence was for condoms, most of the research had been conducted in high-income countries, and analyses provided minimal evidence on the relationship, in any direction, between self-care and reproductive empowerment. Furthermore, even though measures exist to assess reproductive empowerment-related concepts, including scales validated in Kenya, Nigeria, Zambia, Ethiopia, Uganda, and the United States, these were not used in the studies eligible for inclusion in the review.^4^^–^^9^

Understanding the relationship between self-care and empowerment is necessary to improve the design, implementation, and scale-up of family planning self-care interventions.

Scales are^11^^,^^12^:

*measurement instruments that are collections of items combined into a composite score … intended to reveal levels of theoretical variables not readily observable by direct means.*

Family planning programmers and researchers are often interested in measuring constructs such as attitudes, self-efficacy, or empowerment that are not directly observable to determine if their program objectives are being met or why there might be gaps in meeting their objectives. Further, using the same scale to measure a construct across populations, geographies, and/or contexts fosters comparability and produces generalizable knowledge about that construct. The goal of this commentary is to garner support from the family planning community to use existing, validated reproductive empowerment scales to generate comparable evidence and answer questions such as, “Are family planning self-care interventions empowering?” and, “Are self-care interventions more readily used by those who feel more empowered?” Here, we summarize the scales and the results of our thematic analysis to help programmers and researchers select the most appropriate scales to inform their family planning self-care programming.

## THE FRAMING OF REPRODUCTIVE EMPOWERMENT

A place to start understanding reproductive empowerment is by defining empowerment itself. Kabeer suggested a definition of empowerment that is commonly used^12^:

*the expansion in people's ability to make strategic life choices in a context where this ability was previously denied to them.*

According to Kabeer, the core conditions of empowerment are agency, resources, and achievement.^11^ Reproductive empowerment is a type of empowerment that is specific to the life domain of reproduction, including contraception; other types of empowerment in different life domains include economic, legal, and social.

Several frameworks related to reproductive health and empowerment have been developed, and they focus on dimensions such as individual and structural power dynamics, as well as psychosocial processes, beginning with the existence of choice and progressing to the exercise and achievement of choice.^8^^,^^13^^–^^17^ Despite these variations, reproductive empowerment is generally conceptualized as the result of the interaction between individual and structural factors.^12^ For the systematic review on reproductive empowerment and contraceptive self-care, we used the Reproductive Empowerment Framework developed by the International Center for Research on Women (ICRW) with funding from the U.S. Agency for International Development and in partnership with MEASURE Evaluation.^2^ The ICRW framework adopts Kabeer's conditions of agency and resources as its own core components.

Reproductive empowerment is generally conceptualized as the result of the interaction between individual and structural factors.

**Agency,** at the center of the ICRW's Reproductive Empowerment Framework, is defined as individuals' capacity to take deliberate actions to achieve their reproductive goals. The framework describes 3 levels of agency: individual, immediate relational, and distant relational. Individual agency includes comprehensive knowledge, physical and mental health, self-efficacy, and critical consciousness. Immediate relational agency includes characteristics of relationships such as emotional intimacy, communication quality, respect for bodily integrity, and social support. And thirdly, distant relational agency includes resources such as the political, legal, and policy environments, health system culture; gender and reproductive norms; and the physical, cultural, and economic environments. In this framework, **self-efficacy** is considered a “resource” for empowerment.

According to ICRW,^2^

*Resources are “enabling factors” that may act as catalysts for empowerment within the context of specific relationships.*

Agency and self-efficacy are integral components to those interested in measuring and increasing reproductive empowerment in programs and research.

**Reproductive autonomy**, another construct of interest to those considering reproductive empowerment, has been defined by Upadhyay et al. as^4^:

*having the power to decide about and control matters associated with contraceptive use, pregnancy, and childbearing.*

The authors further note that “reproductive autonomy is one domain within the overarching construct of women's empowerment,” which is defined as “the expansion in women's ability to make strategic life choices where this ability was previously denied them,” citing the Kabeer definition. With this framing of reproductive empowerment in mind, we searched for measures that aligned with this framing and the conditions of agency, resources, and autonomy.

## MAPPING MEASURES OF REPRODUCTIVE EMPOWERMENT

We searched the peer-reviewed literature to identify existing scales that measure reproductive empowerment and understand how reproductive empowerment is conceptualized within those scales. We examined references of the articles from the systematic review previously mentioned and searched for related articles using PubMed and Google Scholar. This was not a systematic literature review. We identified 5 validated scales related to reproductive empowerment that may be of interest to family planning programs (Table)^5^^–^^10^^,^^18^: The Reproductive Autonomy Scale, the Reproductive Empowerment Scale, the Contraceptive Self-Efficacy among women in sub-Saharan Africa (CSESSA) scale, Women's and Girls' Empowerment in Sexual and Reproductive Health (WGE-SRH) index, and Sexual and Reproductive Empowerment Scale for Adolescents and Young Adults. The first 4 scales focus on women of reproductive age, and the last scale focuses on youth (aged 15–24 years) of both genders.

**TABLE.** Landscape of Validated Scales for Reproductive Empowerment

Name of Scale and Year Developed	Description	Number of Items and Subscales	Context and Validation	Scale Use and Interpretation
Reproductive Autonomy Scale, 2014^4^	Assess a woman's interpersonal power over reproductive matters including contraception use, pregnancy, and childbearing.	The full scale has 14 items and includes 3 subscales (decision-making index, coercion subscale, and communication subscale).	Developed and validated with women ages 15–60 years in family planning and abortion facilities across the United States.^3^ The construct validation study analyzed the subscales association with unprotected sex. The decision-making subscale was not significantly associated with unprotected sex, but the odds ratio was in the expected direction of higher decision-making capacity associating with lower levels of unprotected sex. The communication and coercion subscales were significantly associated with unprotected sex within the past 3 months in an inverse direction.	Assign scores for each of the 3 subscales: decision-making index (my partner=1; both me and my partner=2; me=3); coercion subscale (strongly disagree=4; disagree=3; agree=2; strongly agree=1); communication subscale (strongly disagree=1; disagree=2; agree=3; strongly agree=4). Sum the scores for each of the 3 subscales. A higher score indicates higher levels of reproductive autonomy.
Reproductive Empowerment Scale, 2019^5^^,^^6^	Assess a woman's ability to make reproductive choices about contraception and sexual relations at interpersonal and community levels.	The full scale has 20 items and includes 5 subscales (RH health care provider communication, RH partner communication, RH decision making, RH social support, and RH social norms).	Developed in the United States and Zambia and validated in Kenya with women aged 15–49 years and men aged 18–59 years and in Nigeria with women aged 18–35 years and male partners of any age.^4^^,^^5^ The construct validity of the subscales was tested for association with the following variables: currently doing something to prevent pregnancy; currently using a method of modern contraception; and likely to use a modern method of contraception in the future. The full scale and subscales were associated with all 3 variables.	Assign scores for the 4 Likert responses (strongly disagree=1; disagree=2; agree=3; strongly agree=4). Sum the scores for each subscale, except for item numbers 13 and 14, and divide the sum by the number of items in the subscale(s). A higher sum represents greater empowerment. Items 13 and 14 will depend on the perception of empowerment in the context of implementation. The authors give this example: “If joint decision making is considered most empowering and decision making by non-partners is considered least empowering, one option for scoring is 4=my partner and myself jointly; 3=myself; 2=my partner; 1=all other options.”
CSESSA scale 2018^7^	Assess a woman's certainty in her ability to initiate, manage, and continue use of contraception.	The full scale has 11 items for Kenya and 10 items for Nigeria within 3 subscales (husband/partner communication, choosing and managing a method, and provider communication).	Based on Levinson's Contraceptive Self-Efficacy scale^17^ and validated in Kenya and Nigeria^6^ with women ages 15 and older. The scale and subscales were all significantly associated with the measure of current modern contraception use. The validation study found that the “choosing and managing a method” subscale varied between the 2 country contexts indicating that the subscale might be more relevant in contexts with low contraceptive prevalence compared to high.	Response options range from 0–10 with 0=cannot do at all and 10=highly certain can do. Sum the scores for each subscale and divide by the number of items in each scale. A higher score indicates higher levels of contraceptive self-efficacy.
WGE-SRH index, 2018^8^	Assess women's constraints and motivations related to the existence of choice and exercise of choice to have sex, use contraceptives, or become pregnant.	The full index has 22 items in 2 subscales (existence of choice and exercise of choice).	Developed and validated in Ethiopia, Nigeria, and Uganda with women aged 15–49 years.^7^ Construct validity for the scale and subscales were tested against measures of volitional sex and contraceptive use. The subscales were associated with the measures for most sites, but there was an unexpected inverse association in Kano State (Nigeria) between exercise of choice and volitional sex.	All items are scored 1 (strongly disagree) to 10 (strongly agree). Sum the scores for all items. The scale authors suggest using additive scores since the measures are meant to indicate “the level of women's SRH empowerment at the population level.” Higher scores indicate higher levels of sexual and reproductive health empowerment.
Sexual and Reproductive Empowerment Scale for Adolescents and Young Adults, 2019^9^	Assess a youth's sexual and reproductive autonomy through self and social support, bodily safety, and sexual pleasure.	The full scale has 23 items in 7 subscales (comfort talking with partner; choice of partners, marriage, and children; parental support; sexual safety; self-love; sense of future; and sexual pleasure).	Developed and validated in the United States with adolescents and young women and men aged 15–24 years.^8^ The scale and subscales were associated with sexual and reproductive health information and access to sexual and reproductive health services measured at baseline and moderately associated with the use of desired contraceptive methods at 3-month follow-up.	The total score ranges from 0 to 92. Response options for each question range from not at all true=0, a little true=1, moderately true=2, very true=3, and extremely true=4. Sum the scores for each subscale. A higher score indicates higher level of sexual and reproductive empowerment. The subscales are independent of each other, so they can be used on their own or the entire scale can be used as a composite measure.

Abbreviations: CSESSA, Contraceptive Self-Efficacy among women in sub-Saharan Africa; RH, reproductive health; RH, reproductive health; WGE-SRH, Women's and Girls' Empowerment in Sexual and Reproductive Health.

**FIGURE 1:**
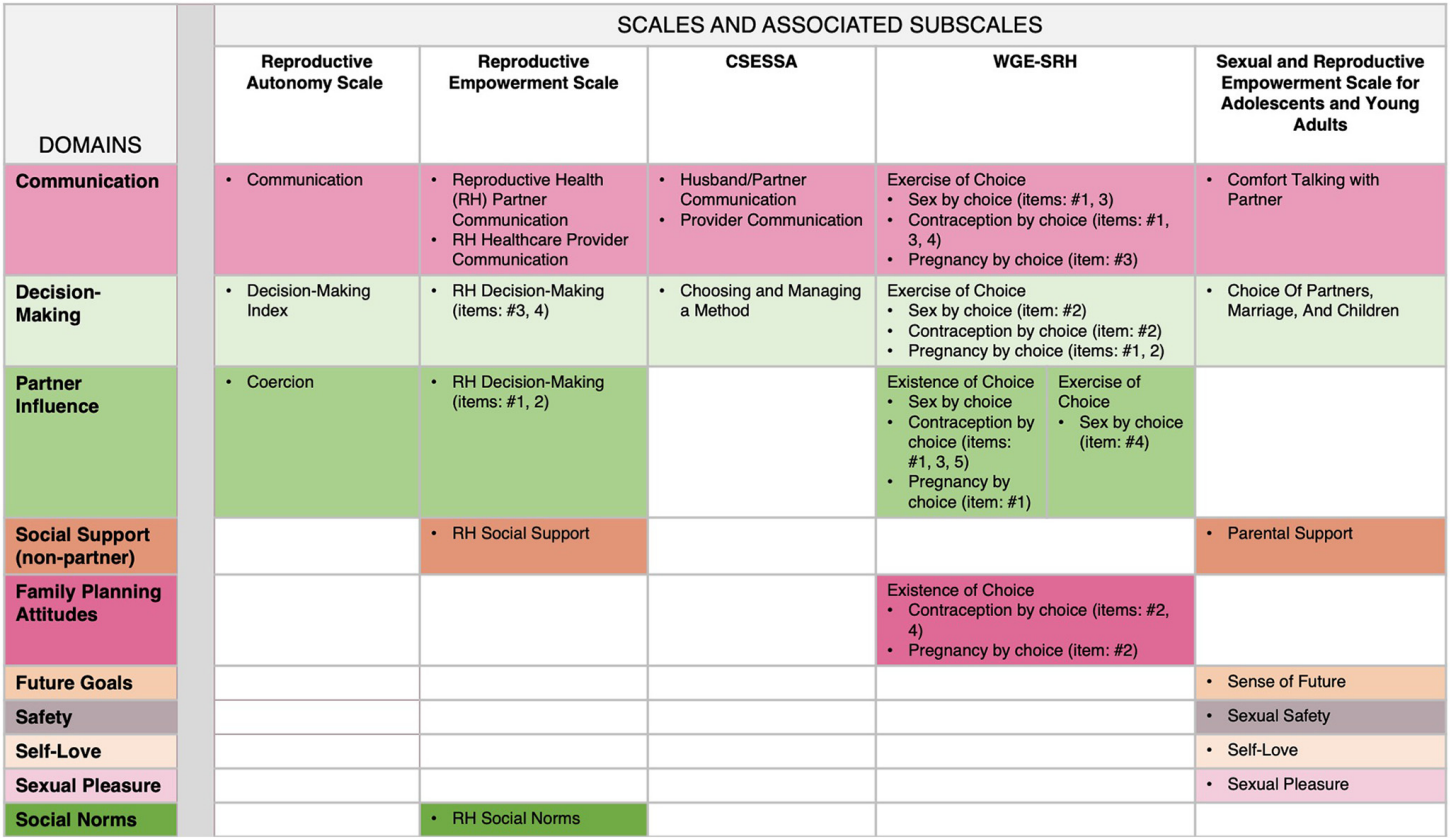
Map of Validated Reproductive Empowerment Scales by Domain Abbreviations: CSESSA, Contraceptive Self-Efficacy among women in sub-Saharan Africa; RH, reproductive health; WGE-SRH, Women's and Girls' Empowerment in Sexual and Reproductive Health.

After examining the existing reproductive empowerment-related scales, we observed thematic similarities across the scales' items even though the authors of the scales used different labels for their constructs. For example, some scales may label an item as “choice” while another scale may label a similar item as “communication.” Programs that seek to identify activities to increase their clients' reproductive empowerment and measure change in reproductive empowerment after implementing these activities may face inconsistencies in terms and definitions. This led us to create a map by thematically grouping the items within the 5 reproductive empowerment-related scales into domains that could be the focus of reproductive empowerment activities within family planning programs (Figure 1).

We mapped the items in the existing reproductive empowerment scales by theme into 10 domains.

We first mapped the domains using the scale authors' labels (not shown). This resulted in 10 domains: (1) communication; (2) decision making; (3) choice (of method, of management of method, and of partner); (4) coercion; (5) social support; (6) future goals; (7) safety; (8) self-love; (9) sexual pleasure; and (10) social norms.

Next, we reviewed the content of the items within each domain (as described by the scale authors) to identify similarities or differences between the items. We also considered whether the current labels reflected the content of the items when looking across the scales. We observed that 3 of the scales had items that measured the influence of sexual partners, and therefore, we grouped the items into a “partner influence” domain. This new domain included the items from the coercion subscale of the Reproductive Autonomy Scale, items #1 and #2 from the RH decision-making subscale of the Reproductive Empowerment Scale, and all 4 items under WGE-SRH's existence of choice, sex by choice subscale, as well as contraception by choice items #1, #3, and #5, pregnancy by choice item #1, and exercise of choice, sex by choice item #4. When we looked at the items scale authors put under the “choice” domain, we noticed they were heterogeneous. We redistributed these items thematically into the existing decision-making and communication domains and the new partner influence domain. Specifically, for the WGE-SRH under exercise of choice, sex by choice item #2, contraception by choice item #2, and pregnancy by choice items #1 and #2 mapped onto the decision-making domain. Also, CSESSA's choosing and managing a method subscale and the Adolescent and Young Adults scale's choice of partners, marriage, and children mapped on the decision-making domain. Several WGE-SRH items under exercise of choice mapped onto the communication domain: sex by choice items #1 and #3; contraception by choice items #1, #3, and #4; and pregnancy by choice item #3. There were a few items from the WGE-SRH (contraception by choice items #2 and #4 and pregnancy by choice item #2) that did not fit in other domains, and we grouped these under a new domain called “Family planning attitudes.” Thus, we ended the thematic process with the following 10 domains and definitions.

1. Communication: Any item indicating an exchange of thoughts, words, or ideas either verbally or nonverbally by the interviewee to another person or to a group of people
2. Decision making: Any item referring to a potential choice being made by the interviewee about their childbearing; sex; marriage; family planning method use, type, and management; pregnancy; child-rearing; and abortion
3. Partner influence: Any item indicating coercion or consequences by an interviewee's sexual partner over family planning method use, pregnancy, and sex
4. Social support: Any item indicating help given to an interviewee by family, friends, or other people in their community (excluding the interviewee's sexual partner)
5. Family planning attitudes: Any item indicating a personal gain or worry due to the use of family planning methods or spacing between pregnancies
6. Future goals: Any item indicating short-term or long-term plans for the interviewee
7. Safety: Any item indicating the interviewee feeling free of harm
8. Self-love: Any item indicating confidence, self-worth, and ownership of oneself
9. Sexual pleasure: Any item indicating feelings of enjoyment or fulfillment for the interviewee or their partner(s)
10. Social norms: Any item indicating societal values experienced by the interviewee through friends or family

**FIGURE 2:**
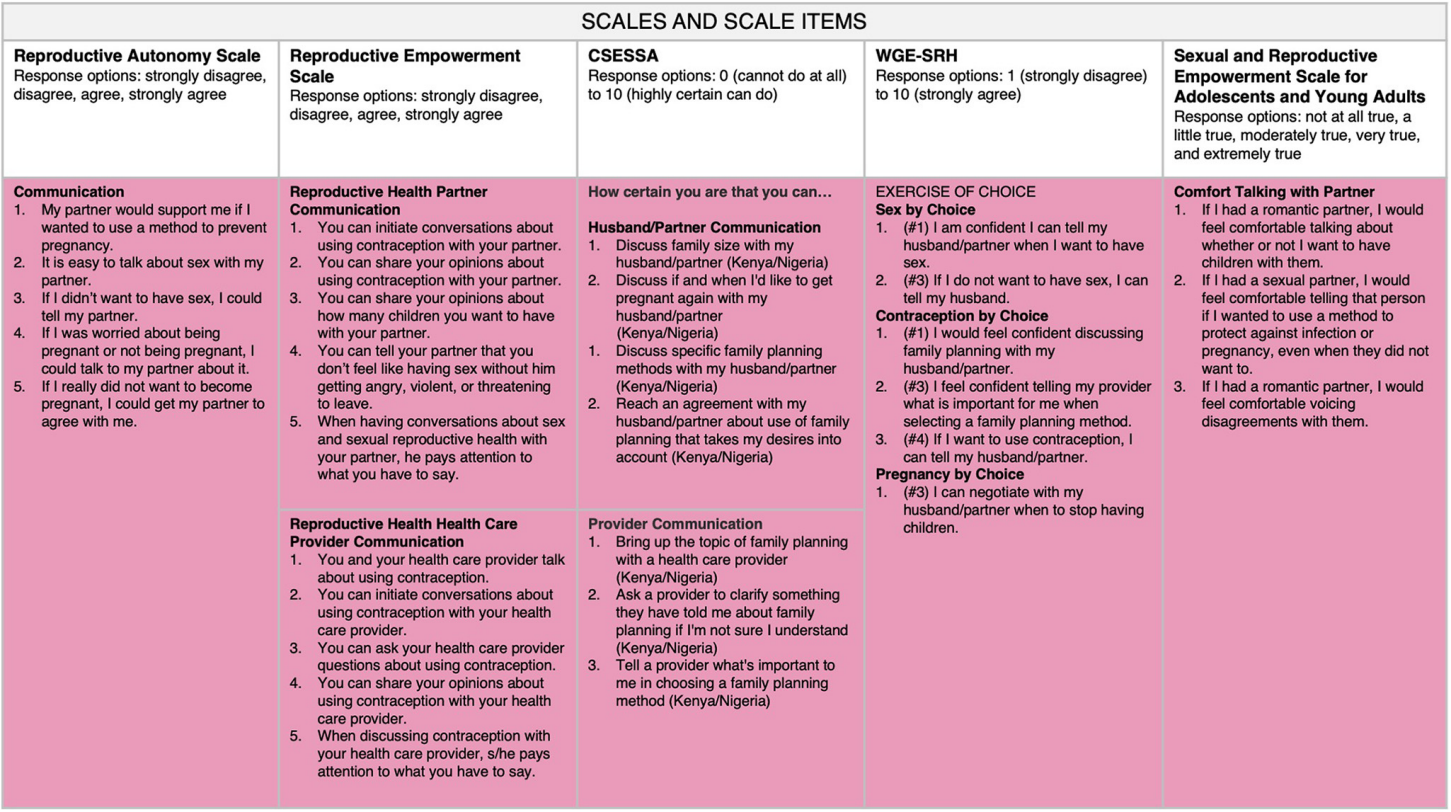
Reproductive Empowerment Scales and Scale Items Within the Communication Domain Abbreviations: CSESSA, Contraceptive Self-Efficacy among women in sub-Saharan Africa; WGE-SRH, Women's and Girls' Empowerment in Sexual and Reproductive Health.

**FIGURE 3:**
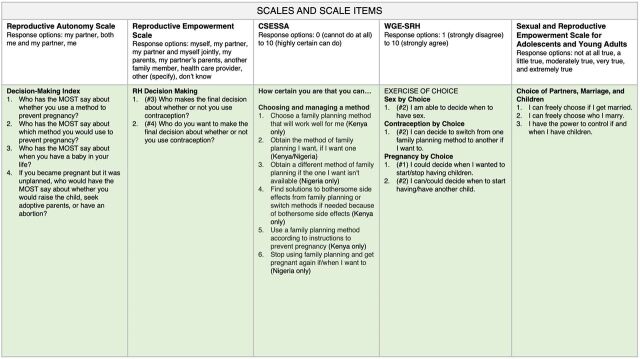
Reproductive Empowerment Scales and Scale Items Within the Decision-Making Domain Abbreviations: CSESSA, Contraceptive Self-Efficacy among women in sub-Saharan Africa; RH, reproductive health; WGE-SRH, Women's and Girls' Empowerment in Sexual and Reproductive Health.

**FIGURE 4:**
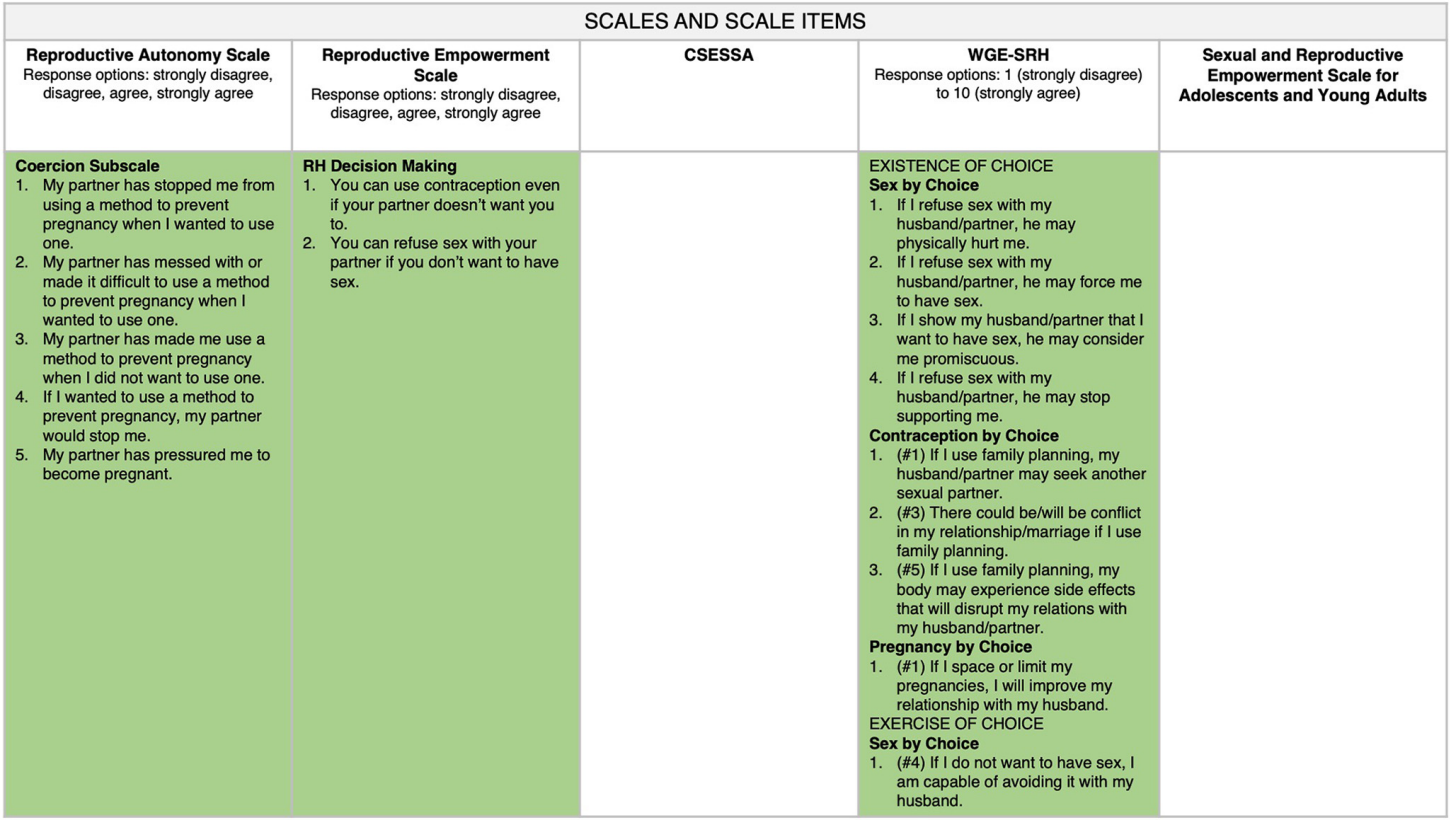
Reproductive Empowerment Scales and Scale Items Within the Partner Influence Domain Abbreviations: CSESSA, Contraceptive Self-Efficacy among women in sub-Saharan Africa; RH, reproductive health; WGE-SRH, Women's and Girls' Empowerment in Sexual and Reproductive Health.

Looking across the scales in our map, we notice that items within the communication domain (Figure 2) and decision-making domain (Figure 3) are represented in all 5 scales and that items from the partner influence domain (Figure 4) are found in 3 of the scales. Items measuring social support (from non-partners) are found in 2 scales, and the rest of the domains are found in only 1 scale. Similar to our findings, a recent systematic review investigating the measurement properties of women empowerment scales in sexual and reproductive health found the most common domains explored were decision making, freedom of coercion, and communication with a partner.^19^

## HOW TO USE THE MAP

Depending on which construct(s) of reproductive empowerment family planning programmers and researchers want to focus on, they may use our map (Figure 1) to identify the scale(s) and/or scale items to measure those specific constructs. The choice of which scales to use should be driven by a context-relevant, clear theory of change. This map serves as a tool to help narrow down the scales based on the program's theory of change. For example, a program or study may be interested in measuring women's agency because they want to determine if self-injectable contraceptive use is empowering. In this situation, they may want to consider using the Reproductive Empowerment Scale because it meets the 3 levels of agency outlined by the Reproductive Empowerment Framework through its 5 domains: individual (partner influence and decision making), immediate relational (communication), and distant relational (social support and social norms) agency. Programs or studies interested in measuring women's contraceptive self-efficacy may want to use the CSESSA because it measures respondents' confidence in performing specific behaviors related to initiating, managing, and continuing use of contraception. However, if items measuring communication are already covered in your questionnaire by another scale, perhaps consider only using the CSESSA subscale “choosing and managing a method” to avoid redundancy and decrease respondent burden. Those interested in measuring autonomy may want to consider using the Reproductive Autonomy Scale or WGE-SRH because of these scales' broad coverage of the topic with the domains of partner influence, communication, and decision making. Finally, the Sexual and Reproductive Empowerment Scale for Adolescents and Young Adults may be useful for programs wanting to measure reproductive autonomy among adolescents.

Family planning programmers and researchers may use the map to identify the scales and/or items to measure specific constructs of reproductive empowerment.

## CONCLUSION

Reproductive empowerment is a broad concept. When measuring reproductive empowerment, there are many existing frameworks, definitions, and scales to draw from. We recommend consistently using existing scales rather than creating new measures or items and measuring specific reproductive empowerment domains. This will allow us to collectively build the evidence base on reproductive empowerment in a way that will advance family planning self-care programming by providing comparable evidence. We mapped the domains and items measured by 5 existing scales. Communication, decision making, and partner-influence are the most measured domains among the scales we examined; however, measures of other reproductive empowerment domains are available and may be of interest to programs depending on their context and focus. Our analysis and mapping may be a useful resource for family planning self-care programmers or researchers, as well as those working in reproductive health in general, who want to focus on certain construct(s) within the broader reproductive empowerment framework.

## Notes

**Peer Reviewed**

**First published online:** June 17, 2022.
